# Treatment of Cancer with Cancer-serum (Erysipelas-serum)

**Published:** 1896-12

**Authors:** 


TREATMENT OF CANCER WITH CANCER-SERUM (ERYSIPELAS-SERUM). Just as a dozen years ago, so to-day, most all physicians are of the opinion that cancer should be classed as an incurable disease. Men like Billroth and Gusseran have acknowledged that they are powerless before this disease; nevertheless, thanks to the efforts of Emmerich and Scholl, the time seems near when the disease will be successfully combated. Following the experiments of Busch, Bruns, and others, who observed that a speedy recovery from carcinoma and sarcoma takes place when the latter are complicated with a concurrent erysipelas, Fehleisen and Neisser injected cultures of erysipelas-cocci in some hopeless cases of cancer, obtaining good results. The use of this means, however, seemed too risky; in one case death resulted. The investigators then had recourse to an erysipelas-serum which contained all the effective ingredients but from which all injurious elements, especially the erysipelas-cocci, had been removed. The method of preparing the serum is as follows: A sheep infected with erysipelas-culture is allowed to bleed to death, and the blood is caught in sterilized glasses. After a definite time the serum is withdrawn in small tubes and then passed through a Chamberlain filter and freed from the cocci. The serum sterilized in this manner is preserved in sterilized tubes holding 10 c.cm., which are sealed with sterilized glue. These tubes are kept in a cool and dark place until used. The experiments with this serum gave striking results a very short time after its application. Only two cases, in which secondary infection, with formation of pus, had taken place, failed to respond. The experimenters think they are justified in regarding this serum as a suitable means of treating cancer. Owing to the fact that the experiments are of very recent date, a number of questions cannot be fully answered, as, for example: Is this serum a specific for all or for only some forms of cancer? In what doses is it to be given? How often must the injection be repeated? Is the use of this serum attended by only good results without dangerous complications? Although in the last experiments no unpleasant effects, even after an injection of 30 c.cm. of serum, were observed, and no rise of temperature could be detected, nevertheless a much greater number of cases must be cited before these questions can be satisfactorily answered (Prof. Emmerich and Dr. Scholl).—*Veeartsenijkundige Bladen*, Deel ix., No. 4.

